# Starting the conversation: community perspectives on preterm birth and kangaroo mother care in southern Malawi

**DOI:** 10.7189/jogh.08.010703

**Published:** 2018-06

**Authors:** Megan Lydon, Monica Longwe, Dyson Likomwa, Victoria Lwesha, Lydia Chimtembo, Pamela Donohue, Tanya Guenther, Bina Valsangar

**Affiliations:** 1 Johns Hopkins Bloomberg School of Public Health, Baltimore, Maryland, USA; 2 Save the Children, Malawi; 3 Johns Hopkins School of Medicine, Johns Hopkins University, Baltimore, Maryland, USA; 4 Save the Children – US, Fairfield, Connecticut, USA

## Abstract

**Background:**

Despite introduction of Kangaroo Mother Care (KMC) in Malawi over a decade ago, preterm birth remains the leading cause of neonatal mortality. Although KMC is initiated in the health care facility, robust community follow-up is critical for survival and optimal development of preterm and low birth weight infants post-discharge. The objective of this qualitative study was to gain insight into community and health worker understanding, attitudes, beliefs and practices around preterm and low birth weight babies and KMC in Malawi.

**Methods:**

A total of 152 participants were interviewed in two districts in southern Malawi, Machinga and Thyolo, in April 2015. Focus group discussions (groups = 11, n = 132) were conducted with pregnant women, community members and women who have practiced KMC. In-depth interviews (n = 20) were conducted with fathers who have practiced KMC, community and religious leaders, and health workers. Purposive and snowball sampling were employed to identify participants. Thematic content analysis was conducted.

**Findings:**

KMC mothers and fathers only learned about KMC and care for preterm newborns after delivery of a child in need of this care. Men typically were not included in KMC counseling due to societal gender roles. Health facilities were the main source of information on KMC, however informal networks among women provided some degree of knowledge exchange. Community leaders were regarded as major facilitators of health information, conveners, key influencers, and policy-makers. Religious leaders were regarded as advocates and emotional support for families with preterm infants. Finally, while many participants initially had negative feelings towards preterm births and KMC, the large majority saw a shift in their perceptions through health counseling, peer modeling, and personal success with KMC.

**Conclusions:**

The findings offer several opportunities to improve KMC implementation including 1) earlier introduction of KMC to pregnant women and their families that are at-risk for preterm birth, 2) greater involvement of men in KMC counselling, practice and care for preterm infants, and 3) strengthening and defining partnerships with community and religious leaders. Finally, as parental perceptions of preterm infants and KMC improved with successful KMC practice, it is hopeful that KMC itself can positively affect social norms surrounding preterm infants, leading to a virtuous cycle of improved perceptions of preterm infants and increased uptake of KMC.

Annually there are approximately 3 million neonatal deaths worldwide [1] of which, more than one-third are related to prematurity [2]. Preterm birth can also have associated morbidities, such as neurodevelopmental impairment, behavioral problems, and a predisposition to chronic disease [3-6]. Kangaroo Mother Care (KMC) is the practice of providing early, continuous and prolonged skin-to-skin contact between the caregiver and her/his preterm or low birth weight baby and exclusive breastfeeding or breast milk feeding. It has been shown to reduce mortality, neonatal sepsis, hypothermia and length of hospital stay, as well as increase exclusive breastfeeding, weight gain, and attachment [7-15]. Important additional features of KMC include early discharge from the health facility with ongoing monitoring and support [16]. Without adequate community follow-up and support, families risk losing any gains in outcomes from facility-initiated KMC. The World Health Organization (WHO) strongly recommends KMC for the routine care of newborns with a birth weight of 2000g or less as soon as they are clinically stable [8].

Malawi has the highest rate of preterm births in the world, estimated to be between 18%-26% of live births [17,18]. KMC was first implemented in Malawi in 1999 and has been scaled up nationally since then, primarily at the hospital level [19]. However, prematurity continues to be the cause of 36% of neonatal deaths in the country [20] and a cohort study conducted in southern Malawi showed that preterm infants were at significantly greater risk of mortality in the first two years of life than at-term newborns [21].

Research conducted in Malawi on attitudes surrounding preterm birth or the care of preterm infants thus far has centered on the cultural language and perception of causes of preterm birth [22,23]. Tolhurst and colleagues provided insight into community perceptions of strategies to prevent preterm births that included prevention and treatment for sexually transmitted infections, avoiding witchcraft, decreasing manual labour, and avoiding violence [22]. Gondwe and researchers explored perceived causes of preterm birth as well as care practices for preterm babies [24]. In two hospitals in Malawi, Chisenga and colleagues assessed mothers’ experiences of KMC and determined that awareness, counselling and support were factors increasing KMC uptake whereas long hospital stay, lack of decision-making power, little support, physical discomfort and preference for incubator care were barriers to uptake [25]. None of these studies, however, examined the wider community perceptions of preterm infants and the practice of KMC. Previous studies have highlighted the important role of social factors in reducing KMC uptake, therefore it is critical to understand community perspectives on these issues [26,27].

Malawi is one of seven countries belonging to the KMC Acceleration Partnership (KAP) Community of Practice. At its inception at the Istanbul KMC Acceleration meeting in 2013, the KAP set a goal of reaching 50% global coverage of KMC by 2020, as part of an integrated reproductive, maternal, newborn, child health package. To reach this goal, the community agreed to nine call-to-action points, including rallying communities and families to support mothers in the practice of KMC and addressing misconceptions and stigma associated with preterm birth, early bonding, skin-to-skin practices, and breastfeeding [28]. This study aims to fill a gap in our understanding of social norms and community perceptions of preterm babies and KMC in a key, high-burden geography, in order to act on the call to action from the Istanbul meeting.

Save the Children-Malawi has initiated a pilot social and behaviour change communication (SBCC) campaign to address attitudes and social norms surrounding preterm birth and KMC. Thus a secondary objective of this research study is to help shape and improve the design of the SBCC campaign by identifying opportunities for targeted messaging and community mobilization activities to improve the survival of preterm and low birth weight babies.

## METHODS

### Study setting

This formative research study was conducted in Machinga and Thyolo districts. Both are located in Southern Malawi, but differ in terms of the population’s tribal affiliation and religion. Machinga is dominated by the Yao tribe and is predominantly Muslim, whereas Thyolo is dominated by the Lomwe tribe, and is predominantly Christian. The study was conducted in these two districts because Save the Children was designing and implementing a pilot SBCC campaign in these districts. In addition, Save the Children had already conducted facility-based KMC strengthening there and wished to leverage existing networks and programmatic efforts to raise awareness and demand for KMC.

In both districts, inpatient KMC services are provided at the district hospital and several other health facilities equipped with maternity units. Other facilities offer ambulatory KMC and referral for those requiring inpatient KMC. When parents deliver a preterm or low birth weight baby at the facility they are admitted to the KMC ward where they receive information about the practice from nurses, nurse-midwives or auxiliary staff. Health Surveillance Assistants (HSAs), who are Ministry of Health employees working at the community level to provide health education and basic preventative and curative health services, are trained to conduct follow-up visits to families of preterm and low birth weight babies.

### Study design

This was a qualitative study in which focus group discussions (FGDs) and in-depth interviews (IDIs) were conducted with participants in the two districts of interest. These included parents already engaged in KMC, pregnant women, community members, health workers and community and religious leaders.

### Sampling

Purposive sampling was used to identify participants; pregnant women were recruited from antenatal care clinics at the district hospitals and community members were recruited from households in the community with the help of district health promotion officers and community leaders. Recruitment for key informants including community and religious leaders, health workers and husbands of women who had delivered preterm babies and practiced KMC, was done with the help of health promotion officers, HSAs, and other district level leaders. Snowball sampling was also used to help identify participants in these categories.

### Data collection

Interviews were conducted in March and April 2015 by a research team led by Save the Children. Two data collectors were recruited from a pool of researchers involved in previous research and trained for two days. Tools were pretested at Bwaila hospital as part of the training. A total of 11 FGDs were conducted for an average length of 75 minutes each (see **Table 1** for full list of interviews conducted). Additionally, 20 IDIs were completed, taking between 30-45 minutes each. Interviews were conducted in Chichewa by the locally trained research team.

**Table 1:** Interviews conducted

Participants and interview type	Sample size (n = 152)
KMC mothers (FGD)	3 groups (36)
Pregnant women (FGD)	4 groups (48); 2 per district
Community members (FGD)	4 groups (48); 2 per district
KMC husbands (IDI)	6
Religious leaders (IDI)	3
Community leaders (IDI)	4
Nurses (IDI)	3
Health Surveillance Assistants (IDI)	4

FGD – focus group discussion, IDI – in-depth interview

The interview guides followed a “funnel approach” whereby participants were asked open, exploratory questions initially, allowing ideas to emerge spontaneously before probing for detailed responses (see **Table 2** for sample questions and Appendices S1-S6 in Online Supplementary Document(Online Supplementary Document) for the full interview guides). All interviews were audio recorded with the consent of participants. Recordings were then transcribed and translated into English by the study team in Malawi.

**Table 2:** Sample of focus group discussion questions

**Selected questions from the FGD guides for pregnant women:**
What do you think about babies who are born before the expected time of delivery? What do people in this community think about these babies?
Have you ever heard about KMC? If yes, what is it? How helpful do you suppose it is? (What do you think about KMC for preterm babies?)
What do you think are the barriers to practicing KMC in your community? (Probe: environmental, cultural, religious, any other)
**Selected questions from the FGD guides for women who have practiced KMC:**
How did you learn to take care of your baby? Where or from whom did you seek care? What happened there?
What do you think about KMC? (Before you tried it? Now if still practicing?) Probe: Do you think KMC is beneficial? Why or why not?
What are the barriers and challenges that you faced/are facing while practicing continuous skin-to-skin care? (Probe about social and cultural context, availability of resources in the facility, privacy, service delivery system, behavior of the nurses and doctors, family support, etc.)

### Ethical considerations

For those who were able to read, the participant was given a copy of the consent form to read. If unable to read, the entire consent form was read to him/her by the interviewer. After review of the consent form, the participant was given time to ask questions for clarification. If the participant agreed to participate in the study, he/she signed the consent form or provided a thumbprint if unable to write.

This study was submitted to both the National Health Sciences Research Council in Malawi and the Johns Hopkins Medical Institutions’ Institutional Review Board, and was granted exemption from review as it was considered non-human subjects research.

### Analysis

Transcripts were uploaded into Atlas.ti and an inductive thematic analysis was employed. Codes were developed through an iterative process by the primary data analyst, whereby transcripts were first read to identify the major themes, which included items such as *source of health education*, *initial reaction to preterm baby*, *community leader* and *religion*. After re-reading through several transcripts, additional codes were created until a coding structure emerged. A final set of codes was finalized after all transcripts were read and all relevant text could be assigned to a code. To analyze the codes by type of participant and study site, a series of matrices were developed so that patterns could be more easily observed and identified. An audit trail was established to document decisions made concerning analysis and interpretation. Results were validated by the in-country research team who provided additional contextual understanding. A central idea that arose in the data was the transfer or dissemination of KMC knowledge and skills to the community, which forms the basis of this article. A subsequent paper will further discuss KMC barriers and enablers at the hospital, community and family levels. The findings presented in this paper are organized by major thematic areas and related sub-categories. Quotes are distinguished by the type of the participant and their regional affiliation.

## RESULTS

### Characteristics of the sample

The majority of KMC mothers, pregnant women, community members, and husbands were subsistence farmers with primary school education. Several participants were small-scale business owners and some women were homemakers. The KMC mothers and pregnant women ranged in age from 17-39 years, with an average age of 25 and were largely married. The husbands ranged in age from 17-60 and the community members were between 25-60 years old. Both community and religious leaders were largely male and married. The religious leaders were all of different denominations.

The HSAs, like the majority of HSAs in Malawi, were males between the ages of 25 and 60 years and resided in one of the villages within their catchment area, an area encompassing between 1000-2000 people [29]. The nurse-midwives were female and had either a diploma or degree from nursing school and were Ministry of Health employees based at either a health center or district hospital.

### Sources of information on preterm birth and KMC

When participants were asked where they received health information, the first source listed by nearly all participants was the health facility. This includes not only pregnant women and KMC mothers but also KMC fathers and community members. Participants also widely agreed that health personnel were their most trusted source of health information. Other sources of health information were noted, including radio, posters and community health dramas, but often only after prompting.

Peer-to-peer information sharing (namely women speaking to other women) was another critical source of health information throughout all interviews. Women would share their experiences of preterm birth and KMC with other women. Despite health facilities and health workers being the primary trusted sources of health information in general, peer-to-peer information sharing was the major source of information on KMC for pregnant women, as none of them had received any information on KMC from the health facility during antenatal care.

“We have not learnt about it, but we do see when it happens in the village that a baby is born preterm. We just hear it from people, but we have not had specific lessons about it” (*Pregnant woman, Thyolo*)

Even recently-delivered women who had received facility-based KMC training spoke extensively of the role other women played in teaching them about KMC.

“My friend’s sister was the one who was helping me and advising me on how to take care of my child since she also has the child who was born before the expected time of delivery” (*KMC mother, Machinga*)

In fact, women expressed a preference to learn from other women who had experienced KMC than from anyone else. In addition, women who had prior experience with KMC were excited by the idea of acting as KMC ambassadors and sharing their experiences more widely.

“There are some women who live very far from hospitals. They give birth to babies and lose hope. If we see such women, we can tell them to attach the baby to the stomach and go to the hospital to receive advice. We can tell them what we did for our babies to grow.” (*KMC mother, Thyolo*)

### Timing of education on KMC

An important aspect of the dissemination of KMC knowledge and skills is its timing, at what point parents or future parents learn about KMC. When asked, all of the KMC mothers and fathers responded that they gained this information at the health facility following the delivery of their preterm baby. While pregnant women made reference to becoming aware of KMC through other women during their pregnancy, parents currently practicing KMC explained that it was only following delivery that they learned the specifics of this practice and were trained on how to do it. The first time parents heard of KMC as a method to care for preterm newborns was during health counseling after birth.

“I didn’t know anything until the time my baby was born” (*KMC father, Thyolo*)

“We had never learnt about it [KMC], but when the babies were born, the doctors taught us about it.” (*KMC mother, Thyolo*)

### Men’s involvement in KMC

Data suggest that men were often left out of the conversation about preterm babies and KMC. Several fathers of low birth weight babies who were receiving or had received KMC felt they had no knowledge of KMC or how to properly care for their newborn. One father described how his wife went to the KMC ward alone and gained instruction from the health staff. It was only later when they were reunited that she could transmit what she had learned to her husband. In a community focus group, members expressed a similar difficulty, highlighting the need for inclusivity of male partners:

“(*Participant 1*) The problem with men is that they don’t go inside the room or ward when their wives or relatives are in the [KMC] ward but instead they stay outside and call their relatives to see them whilst there.

“(*Participant 2*) But some women do not talk or explain about KMC to their husbands.” (*Community members, Machinga*)

As such, it seems that both men and women hold beliefs that the care of newborns rests with the mother:

“Yes, I heard about it [preterm birth], but then we regard these as matters concerning women, that they are the ones who busy themselves with that. But after this happened I realized that it involves us all, that both men and women can play a part.” (*KMC husband, Machinga*)

The traditional societal gender roles establish that men should not play a large role in activities relating to pregnancy, birth and childrearing. Following this, men indicated they were not aware how they can participate or whether their assistance would be accepted. Similarly, some women mentioned feeling uncertain about requesting that their husbands break these social norms. Despite these traditional gender roles, a number of men were accepting of involvement in the care of their preterm infant.

### Role of community leaders

Community leaders, also known as local leaders, are the traditional authority for a community, such as village headman (chief) or group village headman. Local leadership is determined by family lineage, and is not an elected position. When asked about their community’s views on preterm births, all community leaders expressed that preterm infants are “considered people” in their community. At the same time, a couple did caution that “people are still believing in their old traditional culture” (community leader, Thyolo) and that some community members “feel pain or revenge against God” when they have a preterm birth (community leader, Machinga). The local leaders said that they themselves did not discriminate against preterm infants but rather encouraged families, including the men, to care for them. Several leaders expressed concern over the issues they believed caused preterm births including unplanned pregnancies, childbirth at a young age due to child marriage and “God’s will.” In response to these issues community leaders described actions they would like to take including enforcing penalties against those engaging in child marriage, partnering with health workers to promote family planning and counseling on preterm birth, holding community meetings on preterm births and KMC, and creating a network of leaders to better address the issue.

“What is important for the leaders is to come together, to work together and sensitize each other and the people in the community about different programs” (*Community leader, Machinga*)

Participants did not identify community leaders as sources of health education, however in discussions about the role of community leaders it became apparent that they were perceived to play an important role in health education. Community leaders were described as directly providing counseling and encouragement to families with preterm children either through community meetings or household visits. In some communities the chiefs were already engaged in such practices whereas in other communities even if they were not already engaged in these practices, participants suggested this would be a natural role for the community leaders. They were also understood to be facilitators of health education by assembling their communities to receive health messages from other sources.

“They must convene meetings to tell the people about this so that if it happens to anyone, they must attach the baby to their stomach.” (*Pregnant woman, Thyolo*)

“If the chief mobilizes the community, everyone will be present and able to listen and get the information through community drama, songs, poems and the like” (*Female community member, Thyolo*)

The community leaders could also be described as key influencers. They were regarded as having unique power within the community such that knowledge and opinions shared by the chief are readily accepted.

“There is so much that chiefs can do to help the people because whatever the chiefs say, people listen. Chiefs have the final say in all community matters.” (*Religious leader, Machinga*)

One community leader gave an example of utilizing such power whereby he was able to lend legitimacy to the health care system.

“I tell them that when doctors advise them to do something, they have to do it and follow what the doctors advise them.” (*Community leader, Machinga*)

Additionally, community leaders were recognized as policy-makers and rule enforcers. A number of respondents explained that this role could be useful in facilitating KMC. Many gave examples of how chiefs had implemented rules related to improving behavior with respect to safe motherhood and child marriage. They suggested the same could be applied to shift the norms regarding KMC practice among men and general acceptance within the larger community. When asked how a chief could help with KMC, a participant responded:

“By coming up with laws/rules that can help to support KMC practice in the community.” (*Community member, Thyolo*)

Importantly, many participants voiced the concern that all leaders needed to work together on this issue and explained that the community leader was in a position to bring the many parties together.

“Here in [this] village, some people are Christians and others are Muslims. So the chief will call all of us.” (*Community member, Machinga*)

### Role of religious leaders

Participants were also asked about the current and potential role of religious leaders in relation to preterm births and KMC. Some described their religious leaders as “shepherds” who “take interest in where their sheep are going” (community member, Machinga). As such, participants felt they would be conscious of those families undertaking KMC. Other participants described how religious leaders can encourage the family to take care of the preterm baby and assist them with their needs, both spiritual and monetary. The religious leaders themselves saw their role in a similar way, encouraging the families and supporting them through prayer, counselling and helping to raise donations from the community, however they also envisioned acting on behalf of their faith community to request health services from the government and other organizations. While the religious leaders did not describe themselves as educators, this idea was put forward by some participants.

“They have the power to advise people even in churches because a lot of […] women gather there, so the church can offer advice” (*Community leader, Thyolo*)

“They should also encourage their flocks to take care of the preterm babies, they should advise them the advantages of taking care of the children and the good thing about skin to skin care practice” (*Pregnant woman, Thyolo*)

The religious leaders are regarded as community members holding considerable power as well as having a platform through which to share ideas. At the moment however, religious leaders do not consider provision of health information as part of their role. Both Christian and Muslim leaders placed great value on the life of the preterm infant, despite negative cultural associations with preterm infants in Malawi. This view stems from the general value these religions place on life itself:

“The life of every human being begins in the womb” (*Muslim leader, Machinga*)

“On the religious perspective, people believe that a baby born before the expected time of delivery is still God’s creation no matter what” (*Christian leader, Thyolo*)

At the same time, one interview with a Christian leader reflected the conflicting views and complex nature of stigma around preterm birth in the community. While this leader explained that the faith community “rejoices that the church is growing” when a child is born, he soon after noted the common view that “a premature birth is like an abortion, and that is like a curse to us.” Despite this negative connotation, all the religious leaders, including this one, largely spoke of encouraging families to care for the preterm infant and providing spiritual support. The importance of all life was the predominant language used and was reflected in the actions described by these leaders.

### Shifting perceptions

Through the process of learning about KMC and gaining these skills, many parents involved in the practice noted a shift in their perceptions. Initially, many women who practiced KMC said they had been disappointed in having a preterm baby, expressing feelings predominantly of concern, fear and anxiety. The women were worried about survival and how they would care for their baby. Many thought that their newborns were abnormal in their appearance, would not develop like a full-term baby and would quickly die. The fathers of KMC babies had a mixture of positive and negative outlooks on fathering preterm babies. One man described what it meant to him:

“I felt that I was inadequate because the baby was born prematurely […] I was inadequate in that perhaps I was not able to take care of my wife in feeding her, or sometimes disappointing her” (*KMC husband, Machinga*)

Health workers noticed these negative perceptions and described them as a challenge in delivering KMC services.

“These community members gossip and talk bad things about the child when they see the preterm child because they do not know about it” (*HSA, Machinga*)

“It seems they are embarrassed about it. Of course they do not verbalize it, but if you tell them to do this [KMC], they do not do it” (*Nurse, Thyolo*)

Pregnant women were also asked to imagine what it would feel like if they delivered a preterm baby. Two main sentiments arose, a notion of anguish, and the need to reach acceptance.

“In fact it would be painful because this is not how you expected the pregnancy to go, since this is an accident. You do not expect that you will have a preterm baby but that you will have a child who, God willing, will grow into someone you can send on errands.” (*Pregnant woman, Thyolo*)

“I would accept what God has given and take care of the child” (*Pregnant woman, Thyolo*)

There are expectations for a child within the family framework; the child is needed to fulfill certain economic functions and there is a concern that a preterm baby may grow into a weak child who cannot perform these necessary tasks. While several women expressed this concern, some felt it could be mitigated by engaging in KMC:

“If they follow the doctor’s advice, they will go home with their babies, who will grow into exemplary and reliable people who they will be able to send on errands.” (*Pregnant woman, Thyolo*)

While initial reactions to preterm births were negative and most participants doubted the effectiveness of KMC, many saw a shift in their perceptions and related these stories. Perceptions of preterm infants and KMC were modified through three principle mechanisms. The first was through counseling and education provided by health workers, primarily in the health facility: health workers explained the causes of preterm birth and how to perform KMC.

“We learned from the ward where the preterm babies are kept. There they gave us various advice on how to care for the baby, and this encouraged us to follow the advice and realize that the baby was human like any other.” (*KMC woman, Thyolo*)

“Most of them understand but others think it is shameful that we must visit this prematurely born baby. But when we go visiting, we have with us some members of their community who are familiar with them and this encourages such mothers.” (*HSA, Thyolo*)

Second, perceptions were altered by learning about positive examples. Family members who had seen other preterm babies grow up to be strong and healthy children recounted these stories, helping parents develop hope for the future of their own child. In addition, observation of peers at the KMC ward helped shift perception.

“For a mother who follows instructions and feeds the preterm baby well, the baby improves very quickly and becomes an example to other babies who were born preterm at the same time. The other mothers say look, we were together at the preterm ward but look how her baby has grown.” (*HSA, Thyolo*)

Lastly, perceptions were shifted through personal experience. As parents practiced KMC they saw the improvements in their own child, which built confidence in the method and provided positive reinforcement for continuation.

“[My family] also saw that after being cared for and weighed, the baby was improving. This made everyone encourage me to keep the baby attached to the chest.” (*KMC mother, Thyolo*)

“At first I thought that those people who were doing skin-to-skin care were only trying to show off but when I experienced the same situation I saw that it is beneficial.” (*KMC mother, Thyolo*)

## DISCUSSION

This qualitative study addresses a gap in the literature regarding perceptions, attitudes, and beliefs about preterm infants and KMC and highlights potential opportunities to address misconceptions and decrease stigma surrounding preterm birth and KMC.

The findings demonstrate that health facilities are the primary source of information on KMC following delivery, but peer-to-peer information sharing is the primary source for pregnant women, prior to delivery. Men, however, are not included at either time, in part due to societal gender roles. Community leaders were powerful facilitators of health information, health educators themselves, as well as rule-enforcers and decision-makers. Religious leaders are seen as advocates and emotional support to families with preterm newborns. Finally, views of many participants on KMC and preterm infants improved through health counseling at facilities, modeled behavior and personal experience of success with the practice.

Our findings suggest there are missed opportunities prior to the birth of a preterm infant for counseling on preterm birth and KMC. Previous literature shows that earlier awareness of KMC helps increase acceptance of the practice [25]. Antenatal care visits are a key opportunity with a captive audience for discussing KMC, particularly for women who may be at risk for preterm birth. Doing so will require active engagement of health care providers who care for pregnant women, but may not regularly care for newborns. To the best of our knowledge, there are no specific guidelines or operating procedures in Malawi that indicate that KMC should be included in ANC counselling. The National Reproductive Health Service Delivery Guidelines emphasize the use of focused antenatal care, which might assume its inclusion however it does not specify KMC as a part of counseling for women at-risk of preterm birth [30]. Similarly, the Community Based Maternal and Neonatal Health package – Manual for Health Surveillance Assistants emphasizes use of KMC as an intervention for preterm and low birth weight newborns however there is no mention of it regarding ANC [31]. Considering the lack of national policies and guidelines regarding inclusion of KMC, it is understandable that the women in this study reported never receiving KMC counseling prior to giving birth. Further, there is currently no global recommendation to this effect. Given the findings, we recommend that KMC should be included in the internationally recognized standard package of ANC visits for at-risk cases and that the practice of skin-to-skin immediately after birth should be reinforced for all newborns during ANC. This could help emphasize the importance of this practice globally and provide guidance to countries that have not yet integrated the recommendation in their national guidelines. Individual countries however do not have to wait for a global recommendation and we encourage countries to adopt this recommendation in their policies and procedures. In high-burden contexts such as Malawi, we also recommend population-based efforts to shift social norms around skin-to-skin contact, breastfeeding and prematurity in general.

The negative perceptions of preterm births and KMC identified in this study are in line with other research that recognized stigma associated with these experiences [32]. While this stigma has been noted before, this study highlighted that perceptions of preterm birth and KMC were shifted through counseling and practice. This suggests that providing information about KMC earlier in the continuum of care for mothers and newborns may help achieve even better acceptance, preparation, and practice of KMC in the community.

Engagement of community and religious leaders in concordance with their perceived roles in the community can help manage perceptions and beliefs about preterm birth and KMC. Community leaders, for example, seen as facilitators of information, conveners, influencers, and policy-makers, can be engaged by health professionals to reinforce information on preterm birth and KMC and implement programs that support families. Religious leaders, seen as advocates and emotional support for families of preterm and low birth weight babies, can be engaged to provide comfort, community, and solidarity to families, and be a strong voice for particularly vulnerable families that may have trouble navigating the health system or mobilizing resources to care for their preterm infant. Our data also suggest that religious leaders can play an influential role in increasing the value placed on the life of the preterm infant. Other studies reinforce that community and religious leaders can be effective champions of health practices [33,34]. In addition, as informal woman-to-woman education already seems to be the predominant method of learning about preterm birth and KMC, it would be wise to build on this strength by establishing a peer educator model whereby parents who have already experienced KMC can mentor new parents.

Ensuring that KMC is gender-inclusive is critical. Our findings suggest that men do not feel they are considered a key participant in the KMC knowledge and skills acquisition process. KMC can be demanding and time consuming for the caregiver, therefore, full buy-in and participation of both parents is essential for the health of the whole family. To do this, men must be included in the conversation-before, during, and after birth. Our findings do not suggest that men are opposed to participating in care of their preterm infant and KMC training but rather are constrained by societal gender roles. This should be a key target area for social and behavioral change communications. Engaging men in KMC would not only increase shared responsibility for the care of the preterm newborn, it would help shift societal views on the practice. Research shows that male involvement in fields traditionally dominated by women, such as family planning and childbirth, is important for such health practices to gain traction in the community [35,36].

Largely, the results presented here support global findings. As this study highlights a gap in knowledge among new parents beginning KMC, Seidman and colleagues in their systematic review of KMC barriers and enablers, demonstrated that within low and middle income countries, the most significant barrier to KMC adoption among mothers was a low awareness of KMC [37]. Support from family and friends ranked high as an enabler for mothers to conduct KMC and the authors further discussed several studies that highlighted mother-to-mother KMC knowledge sharing. The importance of this peer-to-peer exchange was also identified in the current study. Gender dynamics were not directly listed as a barrier to KMC practice by Seidman et al. however in a more recent systematic review of caregiver perspectives on KMC barriers and enablers, Smith and colleagues discussed it as an important component of the social context that shaped KMC practice [31]. The authors described some fathers “feeling uncomfortable practicing KMC in public [and] learning how to perform KMC while other people were present” while “in some cases, mothers and traditional birth attendants reported feeling uncomfortable with the father performing their duties.” This supports the results drawn from the current study. Seidman and colleagues also highlighted the importance that community may play in LMIC settings on KMC and urged future research to better understand community factors affecting KMC practice. The current study helps fill this gap because unlike many previous studies, the current study not only examines the perspectives of KMC parents and health workers but also of pregnant women, community members, community leaders and religious leaders.

### Limitations

KMC parents included in this study were recruited from KMC wards at health facilities and the pregnant women were recruited from antenatal clinics. As such, the views presented from these groups here are those of people who accessed health care. In addition, interviews were not conducted with parents of preterm or low birth weight newborns who chose not to undergo KMC unless they were included by chance in the community member selection. Inclusion of pregnant women and community members in this study identified barriers to uptake and continuation however future research including non-acceptors of KMC could provide further understanding of barriers to uptake and acceptance. The study design had also planned to capture interviews from a greater number of participants especially among the categories of key informants. This would allow results to be drawn with more confidence. With the present sample, only a few voices provided the basis for comments from community and religious leaders, as well as health workers. Finally, information on participants who declined to participate was not collected.

## CONCLUSIONS

Social norms surrounding preterm birth and KMC can be shifted, and health providers, community leaders, and religious leaders have important roles to play. Timely and opportune introduction of KMC and care for preterm infants, during antenatal care, for example, is needed. There are clear opportunities for better engaging men in the facility-based and community-based care of their preterm infants. KMC requires significant dedication from caregivers, and it is critical that they receive support from their communities if we are to reduce newborn, and particularly preterm mortality, in Malawi and beyond.
