# Supplementary material for: Starting the conversation: community perspectives on preterm birth and kangaroo mother care in southern Malawi
Source: J Glob Health. 2018 Jun 7;8(1):010703. doi: 10.7189/jogh.08.010703 (PMC5993970; doi:10.7189/jogh.08.010703)
Supplement: Online Supplementary Document [file jogh-08-010703-s001.pdf]

Online Supplementary Document

Lydon et al. Starting the conversation: community perspectives on preterm birth and kangaroo mother care in southern Malawi

J Glob Health 2018;8:010703

SNL Malawi SBCC Exploratory Data Collection Tools

Appendix 1: Focus Group Discussion Guide for Pregnant women and Community Members

1. What do you think about babies who are born before the expected time of delivery? What do people in this community think about it?
   Probe
   Do you think they grow up as a normal child or normal adult? why or why not
2. What do you know about the babies who are born before the expected time of delivery?
   Probe
   - Why does it happen?
   - From where did you learn about the babies who are born before the expected date of delivery?
3. What type of care should be given to babies who are born before the expected date of delivery?
4. What does your culture say about babies being born before expected time of delivery?
5. What does your religion say about babies being born before expected time of delivery?
6. How would you feel if your baby was born before the expected time of delivery?
   Probe
   - How would your family feel?
   - What would your neighbors or people in the community say?
7. Have you ever heard about KMC? If yes, what is it? How helpful do you suppose it is? (what do you think about KMC for pre-term babies?)
   Probe
   - Do you think KMC is beneficial? Why or why not?
8. What do you think are the barriers to practicing KMC in your community? (Probe: environmental, cultural, religious, any other)
9. What can facilitate continuation of KMC at home after being discharged fro the health facility? Who can support KMC in the communities?

Probes

- What can families do to help?
- What, if anything, can community members do to help?
- What can HCW (?) do
- What, if anything, can community leaders do to help?
- What can religious leaders do

10. Where do you get health information/advice about pregnancy, delivery, and newborn care from? (PROBE: Radio, Community drama, Posters, Hospitals, HSAs, volunteers, CAGS, care groups, lead mothers)
11. What source of health information trust most? (Radio, HSAs)
12. Have you ever heard of the "Life is Precious" campaign?

Appendix 2: Focus Group Discussion Guide for mothers of who have gone through KMC

1. What do you think about babies who are born before the expected time of delivery?
   Probe:
   - How did you feel when your baby was born before the expected time of delivery?
   - How did your family members feel
   - How did your neighbors and relatives feel
   - What do you think about the future or health of preterm babies?
2. What do you know about the babies who are born before the expected time of delivery? Why does it happen?
   Probe
   - From where did you learn about the babies who are born before the expected date of delivery?
   - Did you know about such issues before your most recent delivery outcome?
3. How did you (learn to?) take care of your baby? Where or from whom did you seek care? What happened there?
   Probe
   - Entire care seeking pathway
   - Who was/has been involved for caring for your LBW baby?
4. Did you take advice from anybody or discussed with anybody about this baby?
   Probe
   - Who did you discuss with
   - What did you discuss about?
   - What did the person tell you or advise you?
5. What do you think about KMC? (before you tried it? now if still practicing?)
   Probe
   Do you think KMC in beneficial? Why or why not?
6. What made it difficult doing/giving KMC in the hospital? How did you overcome the difficulties?
7. What is you experience regarding practicing continuous skin to skin care? How do you feel practicing KMC?
8. Apart from skin to skin care, how else do you keep the baby warm?
9. What support (if any) are/did you receive from your family to practice skin to skin care for your baby? Who supported you?
10. What are the barriers and challenges that you faced/are facing while practicing continuous skin to skin care? (probe about social and cultural context, availability of resources in the facility, privacy, service delivery system, behaviour of the nurses and doctors, family support, etc.)

11. How did you care for your baby once you got home?
12. What is your experience regarding the counselling services? Did you receive any pre-discharge counselling about community continuation of KMC practices?
    Probe:
    - What did they talk about in the counselling session?
    - Did you understand the message? Was it helpful?
    - Was it appropriately delivered? Was it interactive?
    - Please explain and messages or advice?
    - Explore barriers and challenges faced whiling receiving the counselling services?
13. What do you think about the importance of continuing KMC at home once discharged from the health facility?
14. What are the main barriers/ challenges of community continuation of facility based KMC practices? (Probe: Cultural, environmental, economic, etc.)
15. How do take care of your baby (preterm) at home? (probe on the thermal care practice, skin to skin care practice)
16. What is the mechanism of post discharge community follow up of preterm babies after receiving facility based KMC services?

    - Did anybody visit your household to follow up on your preterm baby?
    - Who visited? When was the last time they visited? What did they do during his visit?

17. Who (Does anyone?) supports/encourages mothers to practice KMC?
    Probe

    - At facility
    - At home
    - The role of HSAs and community midwives in community KMC
18. What would you suggest to improve KMC services? To make KMC easier at home?

19. What do you think of a KMC peer mentoring model? Would you want to be supported by another woman who has practiced KMC? Why? Why not?
20. Where do you get health information from?

    - Radio
    - Community drama
    - Posters
    - Nurses, doctors
    - HSAs, volunteers
    - CAGS, care groups, lead mothers

21. What is your most trusted source of health information?
    Probe

- Radio
- HSAs

22. Have you ever heard of the "Life is Precious" campaign?

Appendix 3: In-depth Individual Interview Guide for HSAs and community midwives

1. What do you think about babies who are born before the expected time of delivery?
2. Have you ever come into contact with these preterm newborns and what was the experience like?
3. Can you please give an idea about your responsibilities as an HSA/Community midwife (Probe: try to know how much S/he aware about his/her responsibility & build rapport by this question)
4. Did you receive any training on KMC? (Probe content of the training)
5. Did you receive any training on post discharge community follow up of preterm babies after receiving facility based KMC services? Was the training adequate? Why or why not?
6. What is the mechanism of post discharge community follow up of preterm babies after receiving facility based KMC services?
7. What is your role in it?
8. How do you conduct the follow up visits? Do you follow a specific plan? (Probe: if yes, detail of the plan).
9. What services do you provide during the follow up visits? (Probe on the follow issues)
    a. Counselling on thermal care (skin to skin care): Duration, process, etc
    b. Counselling on feeding
    c. Weight monitoring
    d. Counselling of family members
10. How do you document and report your services related to post discharge community follow up of preterm babies?
11. What are some of the problems that you face when providing KMC services?
12. What do think about the necessity of community continuation of facility based KMC services? (probe on the importance of skin to skin care practice, feeding practice, close monitoring of the baby, etc.)?
13. What do think about the necessity of post discharge community follow up visits to the household of preterm babies? (why important or why not)?
14. What do the mothers & family members think about KMC? (Probe: acceptance, shyness, discomfort, environmental barriers)
15. What are the main barriers/ challenges of community continuation of facility based KMC? (Probe: Cultural, environmental, economic, etc.)
    a. Community continuation of KMC practices
    b. Post discharge community follow up mechanism
    c. What role do you play involvement in supporting KMC once the mothers are discharged?
16. Do you have any and suggestion of recommendation regarding this?
17. How best can we reach women who have gone through KMC? Which communication channels/materials/teaching aids would be useful? of using videos or MP3)

Appendix 4: In-depth Individual Interview Guide for Facility-based nurse midwives

1. Tell me your experience with babies who are born before the expected time of delivery?
2. What services are available in your facility for preterm births and related complications?
3. What are your regular responsibilities? (Probe: routine work, duration/hours of work, details)
4. How important is KMC for preterm babies? (Probe: why/ why not, how it will help, Facility & community based KMC)
5. What is your regular responsibilities regarding KMC service? (Probe: Detail activities related to KMC service)
6. Did you get any training on KMC? (Probe: if yes, details; if no, necessity of training) What do you feel about the training? Was it adequate? Why or why not?
7. What are the challenges that you face helping mothers implement KMC? (probe on continuous skin to skin care practice, alternate thermal care practice, family support for the mothers, hospital environment, etc.)
8. What is the counselling process for mothers receiving KMC services in this UHC?
9. What are challenges that you face during conducting the counselling session? (probe on preadmission counselling, regular counselling, hospital environment, content of counselling, response of the mothers, family members, etc.)
10. What do the mothers & family members think about KMC? (Probe: acceptance, shyness, discomfort, environmental barriers)
11. What other factors affect KMC implementation/adoption in the facility? (Probe: resistance from healthcare providers, availability of resources, false ideas of KMC, family issues, financial burden for family, etc.)
12. Do you have any recommendation to improve the KMC services in your facility? (probe on the following issues)
    a. Counselling
13. What are the main barriers/ challenges of continuing community based KMC? (Probe: Cultural, environmental, pouch, any other)
14. How best can we reach women who have been discharged from the KMC unit KMC? Which communication channels are relevant? (probe feasibility of using videos or MP3 for facility discussions)
15. 15. What kind of job aids/teaching tools would be useful to you in educating mothers and their families about KMC?

Appendix 5: In-depth Individual Interview Guide for Community and Religious Leaders

1. What do people in this community think about newborn babies
   Probe
   Are newborns considered as people?
2. What do people in this community think about babies who are born before the expected time of delivery?
   Probe
   - Why do they think it happens?
   - What do they think are the probable consequences?
3. What does your religion/culture say about babies being born before expected time of delivery?
4. What does your religion say about babies being born before expected time of delivery?
5. Do people think such babies require special attention or different types of care? Why or why not?
6. What do you think is the role of religious leaders in promoting newborn health?
   Probe
   - What can families do
   - What can community members do
   - What can HCW do
   - What can community leaders do
   - What can religious leaders do
7. Have you ever heard of the "Life is Precious" campaign?

Appendix 6: In-depth Individual Interview Guide for Husbands (Fathers of LBW babies)

1. How do you feel being a father of a preterm or low birth weight babies?
2. How did your neighbors and relatives feel when your baby was born before the expected time of delivery?
3. What do you know about the babies who are born before the expected time of delivery? Why does it happen? What do you think his/her future will be like?
4. Where did you learn about babies who are born before the expected date of delivery? Did you know about this before your most recent delivery outcome?
5. How do you think these babies should be cared for? Why or why not?
6. How did you or your family take care of the baby?
7. What role did you play in caring for the baby? (Probe willingness to put baby in KMC position)
8. Where do you get health information from? (Radio, Community drama, Posters, Hospitals, HSAs, volunteers, CAGS, care groups, lead mothers
9. What is your trusted source of health information? (Radio, HSAs)
10. Have you ever heard of the "Life is Precious" campaign?
